# Supplementary material for: Hepatic Steatosis and Diet in Adult Celiac Disease: A Cross-Sectional Study
Source: Nutrients. 2025 Nov 15;17(22):3577. doi: 10.3390/nu17223577 (PMC12655135; doi:10.3390/nu17223577)
Supplement: Supplementary file 1 [file nutrients-17-03577-s001.zip › nutrients-3936496-supplementary.pdf]

Supplement

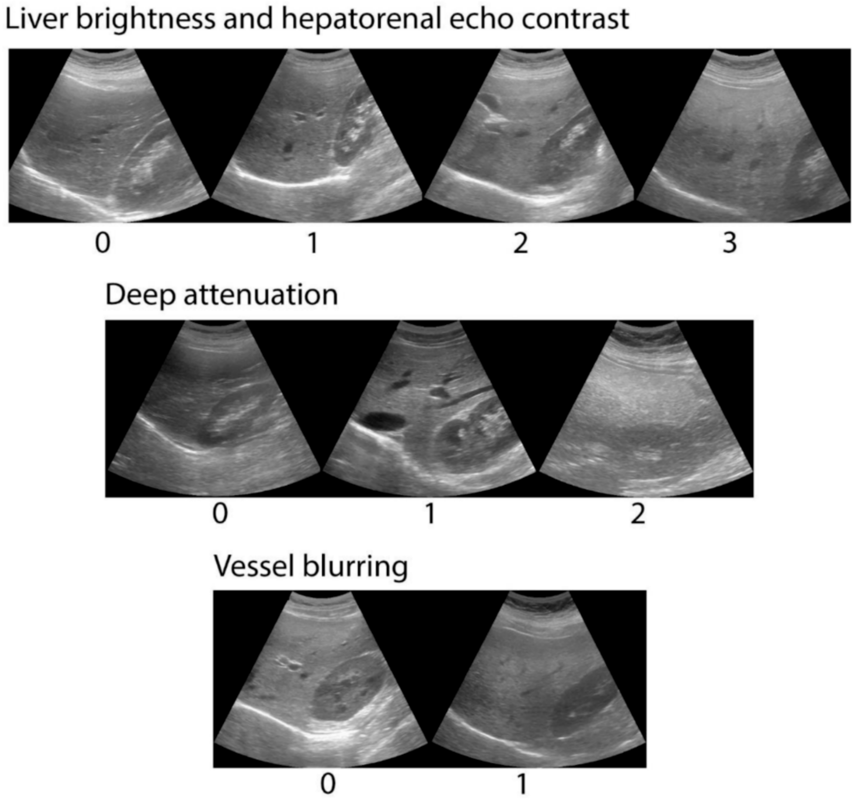

Figure S1. Examples of abdominal ultrasound images combined with the semi-quantitative score system by Hamaguchi et al. to assess liver fat infiltration in Chilean young adults. The system scores liver brightness from 0 to 3, diaphragm attenuation from 0 to 2, and vessel blurring from 0 to 1. The maximum score possible was 6.

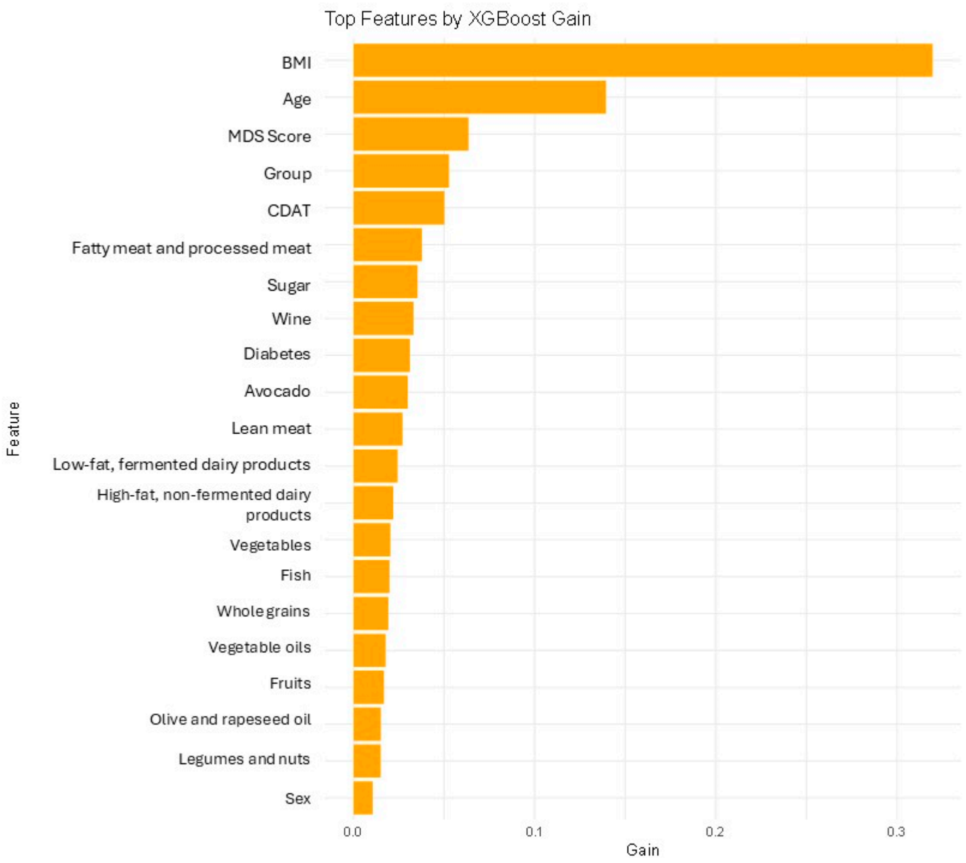

Figure S2. Results of the machine learning analyses.

Table S1.

ID Number: ________

MEDITERRANEAN DIET SCORE (MDS) QUESTIONNAIRE

	Score Items	Units	1 Point	0.5 Point	0 Point
1.	Vegetables (without potatoes)	Servings per day	≥3	1-3	<1
2.	Legumes and nuts	Portions per week	≥2	1-2	<1
3.	Fruits	Servings per day	>2	1-2	<1
4.	Whole grain cereals	Servings per day	≥2	1-2	<1
5.	Lean meat	Servings per week	>4	2-4	<2
6.	Fish	Servings per week	>2	1-2	<1
7.	Fatty meat and processed meat	Servings per week	<1	1-2	>2
8.	High-fat, not fermented dairy products	Servings per day	<1	1-2	≥2
9.	Low-fat and fermented dairy products	Servings per day	≥2	1-2	<1
10.	Vegetable oils	Teaspoons per day	4-8	2-4	>8 or <2
11.	Olive and rapeseed oil	Teaspoons per day	>3	1-3	<1
12.	Avocado	Units per week	>3	0,5-3	<0,5
13.	Sugar	Teaspoons per day	<4	4-8	>8
14.	Wine	Glasses per day	Women: ≤1<br>Men: ≤2<br>Moderate and usually with meals	Women: ≤1<br>Men: ≤2<br>Moderate and rarely with meals	Women: >1<br>Men: >2<br>or non-drinkers

Table S2. Differences in parameters based on the severity of HS. p-values indicate a difference across the groups.

		No HS	Mild	Moderate	Severe	Mild vs. Moderate	Mild vs. Severe	Moderate vs. Severe	No vs. Mild	No vs. Mod.	No vs. Sev.
								p-value			
**Group** (n)	**New CD patients**	30 (16%)	25 (33%)	4 (22%)	1 (17%)	*0.3*	*0.5*	*0.5*	***<0.001***	*0.8*	*0.2*
	**CD patients on a GFD**	102 (53%)	40 (53%)	9 (50%)	1 (17%)						
	**Controls**	59 (31%)	10 (13%)	5 (28%)	0 (0%)						
**Age at enrolment** mean±SD		30 (25, 41)	35 (25, 49)	43 (29, 53)	55 (49, 61)	*0.089*	***0.003***	***0.049***	*0.060*	***0.002***	***<0.001***
**Sex** n (%)	**Women**	157 (82%)	63 (84%)	14 (78%)	3 (50%)	*0.5*	*0.074*	*0.3*	*0.9*	*0.7*	*0.081*
	**Men**	34 (18%)	12 (16%)	4 (22%)	3 (50%)						
**Diabetes** n (%)		3 (1.6%)	3 (4.0%)	1 (5.6%)	2 (33%)	*>0.9*	***0.042***	*0.14*	*0.4*	*0.3*	***0.007***
**BMI** (continuous) mean±SD		21.5 (20.1, 23.9)	24.8 (21.6, 28.1)	27.8 (25.0, 32.5)	32.3 (30.7, 38.6)	***0.007***	***0.004***	*0.2*	***<0.001***	***<0.001***	***<0.001***
**BMI groups**	**Underweight**	23 (12%)	6 (8.0%)	1 (5.6%)	0 (0%)	***0.028***	***0.006***	*0.5*	***<0.001***	***<0.001***	***<0.001***
	**Normal**	131 (69%)	32 (43%)	2 (11%)	0 (0%)						
	**Overweight**	31 (16%)	23 (31%)	7 (39%)	1 (17%)						
	**Obese**	6 3.1%)	14 (19%)	8 (44%)	5 (83%)						
**MDS** mean±SD		5.50 (4.50, 7.00)	5.00 (4.50, 6.50)	5.00 (4.00, 7.00)	5.75 (5.00, 7.00)	*0.6*	*0.5*	*0.8*	*0.2*	*0.2*	*0.9*
**Vegetables** n (%)	0	24 (13%)	12 (16%)	4 (22%)	0 (0%)	*0.6*	*0.2*	*0.3*	*0.2*	*0.5*	*0.5*
	0.5	134 (70%)	56 (75%)	12 (67%)	4 (67%)						
	1	33 (17%)	7 (9.3%)	2 (11%)	2 (33%)						
**Legumes and nuts** n (%)	0	86 (45%)	36 (48%)	10 (56%)	2 (33%)	*>0.9*	*0.4*	*0.4*	*0.5*	*0.7*	*0.8*
	0.5	84 (44%)	28 (37%)	6 (33%)	4 (67%)						
	1	21 (11%)	11 (15%)	2 (11%)	0 (0%)						
**Fruits** n (%)	0	36 (19%)	16 (21%)	5 (28%)	0 (0%)	*0.3*	*0.5*	*0.11*	*0.8*	*0.12*	*0.7*
	0.5	106 (55%)	43 (57%)	12 (67%)	4 (67%)						
	1	49 (26%)	16 (21%)	1 (5.6%)	2 (33%)						
**Whole grains** n (%)	0	106 (55%)	46 (61%)	7 (39%)	5 (83%)	*0.2*	*0.8*	*0.3*	*0.7*	*0.3*	*0.6*
	0.5	65 (34%)	21 (28%)	8 (44%)	1 (17%)						
	1	20 (10%)	8 (11%)	3 (17%)	0 (0%)						

**Lean meat** n (%)	0	16 (8.4%)	13 (17%)	0 (0%)	0 (0%)	*0.2*	*0.3*	*0.4*	*0.10*	*0.5*	*0.7*
	0.5	109 (57%)	36 (48%)	10 (56%)	5 (83%)						
	1	66 (35%)	26 (35%)	8 (44%)	1 (17%)						
**Fish** n (%)	0	143 (75%)	56 (75%)	11 (61%)	4 (67%)	*0.2*	*0.7*	*>0.9*	*0.9*	*0.2*	*0.7*
	0.5	40 (21%)	17 (23%)	5 (28%)	2 (33%)						
	1	8 (4.2%)	2 (2.7%)	2 (11%)	0 (0%)						
**Fatty meat and processed meat** n (%)	0	45 (24%)	21 (28%)	2 (11%)	1 (17%)	*0.2*	*0.9*	*>0.9*	*0.8*	*0.3*	*>0.9*
	0.5	74 (39%)	28 (37%)	10 (56%)	3 (50%)						
	1	72 (38%)	26 (35%)	6 (33%)	2 (33%)						
**High-fat, non-fermented dairy products** n (%)	0	36 (19%)	11 (15%)	3 (17%)	2 (33%)	***0.037***	*0.11*	***0.011***	*0.7*	***0.010***	*0.2*
	0.5	62 (32%)	28 (48%)	12 (67%)	0 (0%)						
	1	93 (49%)	36 (48%)	3 (17%)	4 (67%)						
**Low-fat fermented dairy products** n (%)	0	81 (42%)	29 (39%)	8 (44%)	1 (17%)	*0.3*	*0.4*	*0.4*	*0.7*	*0.2*	*0.2*
	0.5	81 (42%)	36 (48%)	10 (56%)	5 (83%)						
	1	29 (15%)	10 (13%)	0 (0%)	0 (0%)						
**Vegetable oils** n (%)	0	52 (27%)	22 (29%)	4 (22%)	2 (33%)	*>0.9*	*0.3*	*0.4*	*0.7*	*>0.9*	*0.5*
	0.5	122 (64%)	49 (65%)	13 (72%)	3 (50%)						
	1	17 (8.9%)	4 (5.3%)	1 (5.6%)	1 (17%)						
**Olive and rapeseed oil** n (%)	0	105 (55%)	50 (67%)	15 (83%)	5 (83%)	*0.10*	*0.7*	*>0.9*	*0.10*	***0.044***	*0.6*
	0.5	74 (39%)	24 (32%)	2 (11%)	1 (17%)						
	1	12 (6.3%)	1 (1.3%)	1 (5.6%)	0 (0%)						
**Avocado** n (%)	0	154 (81%)	65 (87%)	15 (83%)	6 (100%)	*0.6*	*>0.9*	*0.5*	*0.082*	*>0.9*	*0.6*
	0.5	36 (19%)	8 (11%)	3 (17%)	0 (0%)						
	1	1 (0.5%)	2 (2.7%)	0 (0%)	0 (0%)						
**Sugar** n (%)	0	22 (12%)	7 (9.3%)	4 (22%)	1 (17%)	*0.090*	*0.8*	*0.8*	*0.7*	*0.2*	*0.9*
	0.5	69 (36%)	24 (32%)	8 (44%)	2 (33%)						
	1	100 (52%)	44 (59%)	6 (33%)	3 (50%)						
**Wine** n (%)	0	114 (60%)	53 (71%)	12 (67%)	3 (50%)	*0.3*	*0.3*	*0.2*	*0.3*	*0.4*	*0.7*
	0.5	42 (22%)	11 (15%)	5 (28%)	1 (17%)						
	1	35 (18%)	11 (15%)	1 (5.6%)	2 (33%)						
**SDE** (Only in CD patients on a GFD)	0	0 (0%)	1 (2.5%)	0 (0%)	0 (0%)	*0.8*	*0.6*	*>0.9*	***0.045***	*>0.9*	*0.5*
	1	51 (50%)	13 (33%)	5 (56%)	3 (60%)						
	2	39 (38%)	18 (45%)	3 (33%)	1 (20%)						
	3	12 (12%)	6 (15%)	1 (11%)	1 (20%)						

	4	0 (0%)	0 (0%)	0 (0%)	0 (0%)	
	5	0 (0%)	1 (2.5%)	0 (0%)	0 (0%)	
	6	0 (0%)	1 (2.5%)	0 (0%)	0 (0%)	

CD: celiac disease, HS: hepatic steatosis, GFD: gluten-free diet, SD: standard deviation, BMI: Body Mass Index, US: ultrasound, MDS: Mediterranean Diet Score, IQR: interquartile range, SDE: Standardized Dietitian Evaluation. Statistical test: Kruskal–Wallis rank sum test, Chi-squared test, Fisher's exact test as appropriate, level of significance: $p < 0.05$. Significant results are highlighted with bold numbers.

Table S3. Binary logistic regression model (Model 2) identifying predictors of hepatic steatosis in celiac patients.

Variable		N	Cases	OR	95% CI	*p*-Value
**Age**		156	54	1.02	0.980; 1.05	*0.381*
**Sex**	**Male**	29	12	1.00	-	
	**Female**	127	42	1.15	0.391; 3.53	*0.806*
**Diabetes mellitus**	**No**	150	51	1.00	-	
	**Yes**	6	3	2.31	0.248; 18.9	*0.431*
**BMI**		156	54	1.30	1.18; 1.46	***<0.001***
**Vegetables**	0	134	49	1.00	-	
	1	22	5	0.310	0,057; 1.32	*0.138*
**Fruits**	0	118	44	1.00	-	
	1	38	10	0.546	0.179; 1.56	*0.269*
**Whole grains**	0	145	48	1.00	-	
	1	11	6	5.50	1.18; 27.5	***0.031***
**Fish**	0	150	52	1.00	-	
	1	6	2	0.781	0.075; 6.02	*0.819*
**Fatty meat and processed meat**	0	105	36	1.00	-	
	1	51	18	1.05	0.363; 2.99	*0.926*
**High-fat, non-fermented dairy products**	0	91	34	1.00	-	
	1	65	20	0.476	0.165; 1.28	*0.152*
**Olive and rapeseed oil**	0	148	52	1.00	-	
	1	8	2	0.481	0.029; 4.25	*0.554*
**Sugar**	0	70	22	1.00	-	
	1	86	32	0.986	0.384; 2.51	*0.976*
**Wine**	0	133	45	1.00	-	
	1	23	9	1.14	0.304; 4.07	*0.976*
**SDE**	**Adequate**	134	44	1.00	-	
	**Inadequate**	22	10	1.15	0.309; 4.03	*0.828*
**MDS**		156	54	1.21	0.811; 1.82	*0.348*

OR: Odds Ratio, CI: Confidence Interval, CD: celiac disease, BMI: Body Mass Index, MDS: Mediterranean Diet Score, SDE: Standardized Dietitian Evaluation, Cases: number of hepatic steatosis cases. Statistical test: binary logistic regression model, level of significance: $p < 0.05$. Significant results are highlighted with bold numbers.
